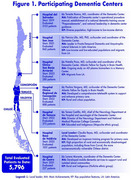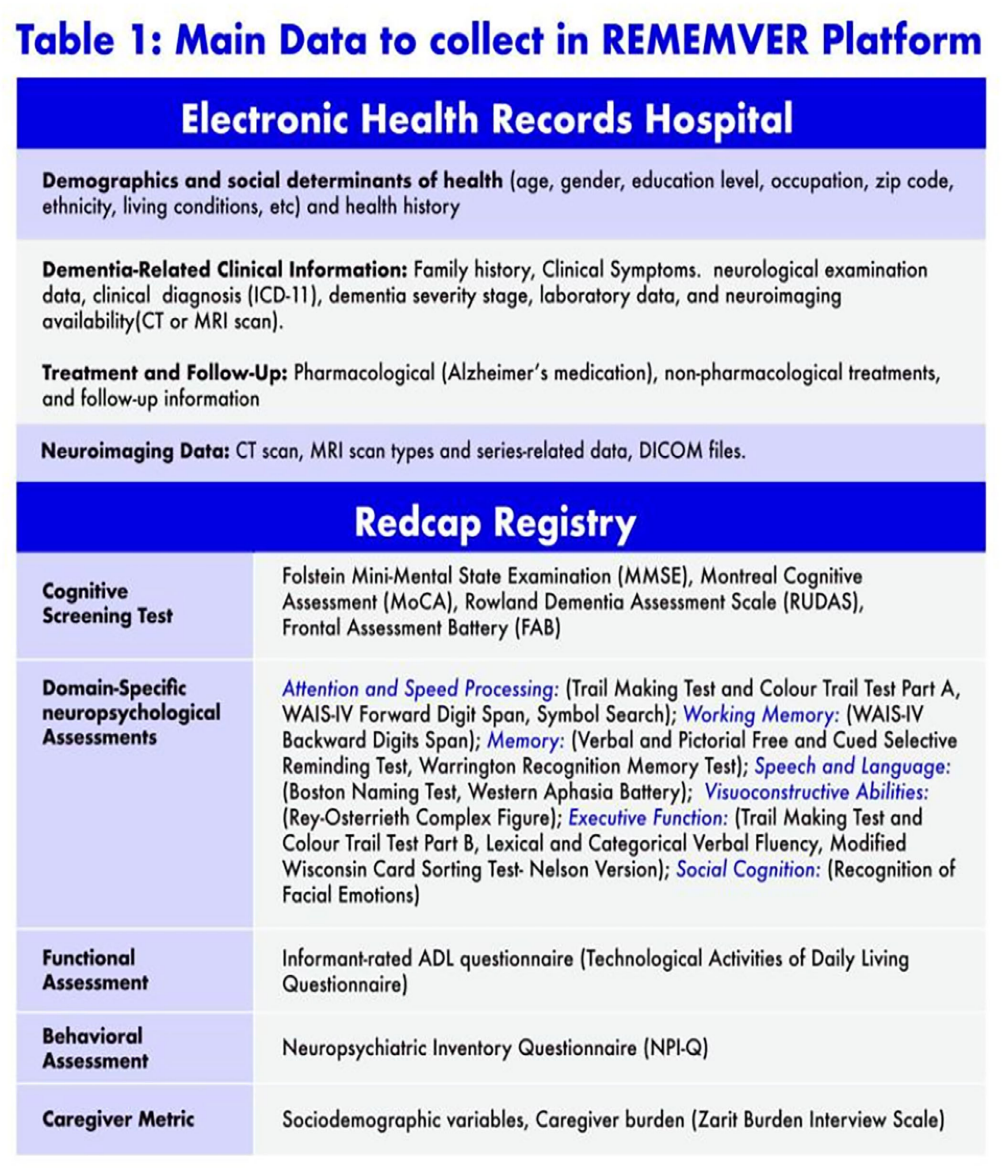# Establishing a Real‐World Data Platform for Dementia Care in Chile: The Red Memoria Viva Electronic Registry “REMEMVER” Initiative

**DOI:** 10.1002/alz70861_108395

**Published:** 2025-12-23

**Authors:** Natalia Pozo Castro, Mauricio Cerda, Paulina Vergara, Ignacio López, Teresita Ramos Franco, Tomas Leon, Carlos Orellana, Pamela Guevara Alvez, Claudia Reyes, Teresa Castillo, Lorena Morante, Francisco Gutiérrez, Patricia Lillo, Andrea Slachevsky Chonchol

**Affiliations:** ^1^ Hospital San Borja Arriarán, Santiago, Region Metropolitana Chile; ^2^ Global Brain Health Institute, Memory and Aging Center, University of California San Francisco, San Francisco, CA USA; ^3^ University of Chile, Santiago, Region Matropolitana Chile; ^4^ University of Chile, Biomedical Neuroscience Institute (BNI), ICBM, Santiago, Metropolitana Chile; ^5^ CIMT Center for Medical Informatics and Telemedicine, School of Medicine, Universidad de Chile, santiago Chile; ^6^ Geroscience Center for Brain Health and Metabolism (GERO), Santiago Chile; ^7^ Global Brain Health Institute, Dublin, County Dublin Ireland; ^8^ Guillermo Grant Benavente Regional Hospital, Concepción, Bíobio Chile; ^9^ Hospital del Salvador & Faculty of Medicine, University of Chile., Santiago Chile; ^10^ Hospital del Salvador, Santiago, Metropolitana Chile; ^11^ Global Brain Health Institute, Dublin, Ireland Ireland; ^12^ Memory and neuropsychiatry disorders Clinic (CMYN), Santiago Chile; ^13^ GBHI, San Francisco, CA USA; ^14^ Biomedical Engineering Department. Universidad de Concepción, Concepción, Bio Bio Chile; ^15^ Geroscience center for mental health and metabolism, Santiago de Chile, Metropolitana Chile; ^16^ Hospital Base de Valdivia, Valdivia, Los Ríos Chile; ^17^ Hospital Clínico Regional de Temuco, Temuco, Araucanía Chile; ^18^ Universidad de la Frontera, Temuco, Araucanía Chile; ^19^ Hospital de Castro, Castro, Los Lagos Chile; ^20^ University of Chile, Santiago, Metropolitana Chile; ^21^ Neurology Unit, Hospital San José, Santiago Chile; ^22^ Department of Neurology South, Faculty of Medicine, University of Chile, Santiago Chile; ^23^ Universidad de Chile, Santiago Chile; ^24^ Memory and Neuropsychiatric Center (CMYN), Neurology Department, Hospital del Salvador and Faculty of Medicine, Universidad de Chile, Santiago Chile; ^25^ Neurology Service, Department of Medicine, Clínica Alemana‐Universidad del Desarrollo, Santiago, Chile., Santiago Chile

## Abstract

**Background:**

Real‐world Data (RWD) is key to advancing research and care on Dementia. Despite having the highest dementia prevalence and a unique and diverse population, there is no dementia registry in Latin America (LA), where the lack of well‐developed specialty centers is a critical barrier to its development. In Chile, a nationwide network of public dementia centers has been implemented, however, despite all of them using electronic health record (EHR), there is no standardized system to integrate the collected data, which limits clinical decision‐making, research, and policy development. To address this challenge, we propose implementing LA´s first dementia RWD platform, “Red Memoria Viva Electronic Registry” (REMEMVER).

**Method:**

Building upon an already existing RWD Registry piloted at the Hospital del Salvador, REMEMVER will expand data integration across seven Chilean dementia centers (Figure 1). Through this project, we plan to standardize clinical, neuropsychological and neuroimaging evaluations; harmonize the collected data aligning with international initiatives (ADNI‐GAIN); develop and implement a RWD platform and establish a dissemination strategy fostering international collaboration. The collected information (table 1) will be recorded in local and central RWD platforms using customized Research Electronic Data Capture (REDCap) instances at the University of Chile, after stablishing automatic integration of the local EHR with the platform. To ensure reliable and standardized data collection across centers, we will develop a hybrid training program to prepare personnel to follow harmonized procedures.

**Results:**

Currently, 5,796 patients have been assessed at the seven participating dementia centers, which supports the institutions capacity to implement the project (Figure 1). We expect that the REMEMVER platform will enhance data standardization and interoperability among healthcare institutions, improve clinical efficiency by reducing waiting times and optimizing patient care‐pathways, and improve Chile’s capacity for dementia research by providing high‐quality, real‐world patient data for epidemiological studies, risk factor analysis, and treatment outcome evaluation.

**Conclusion:**

Through REMEMVER, Chile aims to create a sustainable, regionally integrated RWD platform that enhances dementia care, supports research, informs public health policy and contributes to advancing health equity. This initiative will contribute to global efforts in dementia research by providing unique insights into real‐world patient trajectories in Latin America.